# Approximating the influence of external factors on the technical efficiency score of hospital care: evidence from the federal states of Germany

**DOI:** 10.1186/s13561-022-00414-7

**Published:** 2023-01-25

**Authors:** Iveta Vrabková, Sabrina Lee

**Affiliations:** Department of Public Economics, Faculty of Economics, VSB-Technical University of Ostrava, Sokolská třída 33, 702 00 Ostrava 1, Czech Republic

**Keywords:** Data Envelopment Analysis, Efficiency, Germany, Federal states, General hospitals, Tobit regression

## Abstract

**Background:**

A good health care system and, especially, the provision of efficient hospital care are the goals of national and regional health policies. However, the scope of general hospital care in the 16 federal states in Germany varies considerably from region to region. The objectives of this paper are to evaluate the technical efficiencies of all general hospitals of the 16 federal states for the period from 2015 to 2020, to find out the relation between the exogenous factors and score of efficiency, and also the influence of the COVID-19 pandemic on the results of the technical efficiency of hospital care in the German states.

**Methods:**

A two-step approach was used. First, an input-oriented Data Envelopment Analysis model with constant returns to scale and variable returns to scale was applied for the 6-year period from 2015 to 2020. The calculation of technical efficiency according to the input-oriented DEA model contains the three components—total technical efficiency (TTE), pure technical efficiency (PTE) and scale efficiency (SE). In the second stage, the influence of exogenous variables on the previously determined technical efficiency was evaluated by applying the tobit regression analysis.

**Results:**

Although the level of average technical efficiency of about 90% is high, total technical efficiency deteriorated steadily from 2015 to 2020. Its lowest point at around 78%, was in the year 2020. The deterioration of the average technical efficiency is notably influenced by the lower results in the years 2019 and 2020. The decomposition of technical efficiency also revealed that the deterioration of overall average efficiency was influenced by both pure technical efficiency (PTE) and scale efficiency (SE). Based on the tobit regression analysis performed, it was possible to conclude that the change in the efficiency score can be explained by the influence of exogenous factors only from 6.4% for overall efficiency and from 7.1% for scale efficiency.

**Conclusions:**

The results of the analysis of the overall technical efficiency reveal that the aggregated data of all general hospitals of all 16 federal states show a steadily worsening total technical efficiency every year since 2015. Although, especially, the deterioration of the year 2020 with the occurrence of COVID-19 pandemic, contributes to a deteriorated efficiency average, the deterioration of the efficiency values, based on the analysis performed, is also observable between the years 2016 and 2019. Considering the output generated, for inefficient units and the relevant policy authorities in the hospital sector, it can be recommended that the number of beds and in particular the number of physicians, should be reduced as inputs. Based on this study, it is also recommended that decisions to increase the efficiency of general hospitals should be made with consideration of exogenous factors such as the change in the number of general hospitals or the population density in the respective state, as these had explanatory value in connection with the increase in efficiency values. Due to the wide variation in the size of the federal states, the recommendation is more appropriate for federal states with low population density.

## Introduction

Inefficiency is a pervasive problem in healthcare systems. In fact, the World Health Organization estimates that, on average, 20—40% of total health spending worldwide is wasted [1, 2]. It is therefore not surprising that this political and economic discussion about the efficiency and quality of the public health care system has been going on for many years, not only in Germany but at the international level as well [3–5].

The evaluated German health care system has been the subject of ever increasing political and economic debate for many years. Almost as an alternative, the efficiency of the health care system, the quality of the services offered, and the level of expenditures and costs have been discussed [6–9]. In the discussion about possible cost-saving potential of the various groups of service providers such as physicians, pharmacies and hospitals and their respective associations, the focus on the hospital sector appears to make the most sense. Especially since the hospital sector is regularly named first when it comes to potential cost savings and the issue of cost explosion in the healthcare system [6, 10, 11]. At first sight, this seems understandable, since spending on inpatient hospital services accounts for the largest share of costs in the German health care system in absolute terms [6]. With more than 114 billion Euros, around 25% of total healthcare spending is generated by the hospital sector alone [12, 13].

The range and scope of hospital care is different at the regional and local level of the states. Most providers (hospitals) are located in larger cities and rural regions do not have comparable availability, including the scope of hospital care. This knowledge is valid in many countries of Europe and the World. The key, but not the only, determining differences between regions are the size of the territory and the number of inhabitants [14–16].

Estimation of the technical efficiency of hospital care at the level of regions (states) can be realized using the DEA model and its decomposition. Decomposition allows to detect the importance of returns to scale on the resulting efficiency score [17, 18]. In economic theory, returns to scale belong to classic microeconomic topics associated with the function of production of production units and, under certain assumptions, can be considered a special case of economies of scale. It can therefore be assumed that the effectiveness of the scope of health care viewed in an aggregated perspective, i.e., in the conditions of a certain territorial unit, is influenced by its size. The resulting score of the technical efficiency of hospital care at the level of the individual federal states is determined by selected inputs and outputs, yet the results of the countries' efficiency can be perceived in a broader context. At a minimum, it is necessary to consider the economic character of hospital care as a public good, as well as its social value, and then to deal with the conflict between efficiency and equality. On the other hand, it should be remembered that most state health policies declare improvements in efficiency and equality in health care, even though it is true that to achieve higher efficiency it is necessary to give up a certain degree of equality and vice versa [19, 20].

Generally, hospitals are among the most important providers of healthcare and thus are an essential part of the infrastructure of the economies of developed countries. In the field of hospital care, there are public hospitals, private non-profit and private for-profit hospitals. Many works address whether public or private hospitals are more efficient [21–23], although they always emphasize the limiting factors of efficiency in the form of quality of care. In the last 20 years, it has also been possible to observe social pressure to improve the efficiency of hospital care. As a part of health reforms, the optimization of the bed fund and the reduction of the average treatment time (hospitalization) are being implemented. This is happening in the context of changes in the payment mechanisms for financing inpatient care, new medical options, whether it comes to the equipment of medical facilities, or new patient treatment procedures. Integrated systems of community and home care are being implemented in some medical fields in some countries (e.g., interns, psychiatry) [16, 24].

The question of the efficiency and effectiveness as well as, consequently, the sustainability of the health care system has arisen recently, especially in the context of the COVID-19 pandemic, when governments started to look for solutions, especially for the financing of the health care system in such crisis [12, 25, 26]. The COVID-19 pandemic is a serious health emergency that has therefore affected the lives of everyone around the world [27, 28]. The hospitals are particularly in the centre of the stage [29]. The Corona pandemic COVID-19, which has been emerging since the beginning of 2020, is also confronting the German healthcare system with extreme challenges [12, 30, 31].

Estimation of the technical efficiency of the hospital care is relevant for the reasons mentioned above. The estimation is concluded at the level of the federal states of Germany, including the evaluation of external factors that influence it, and thus fills a gap in research. The paper focuses on the evaluation of the score of technical efficiency of the hospital care in the federal states of Germany for the period from 2015 to 2020, including the approximation of the effect of selected exogenous factors on the score of individual components of technical efficiency.

The research focuses on four research questions (RQs): RQ1: Does pure technical efficiency or scale efficiency affect the overall technical efficiency score of hospital care in the federal states of Germany? RQ2: Can the effect of the COVID-19 pandemic be detected on the technical efficiency score of hospital care in the federal states of Germany?; RQ3: Does the technical efficiency score affect the number of general hospitals in the federal states of Germany?; RQ4: Does the population density in German states affect the technical efficiency score?

### Literature review

While studies on efficiency measurement using the DEA method with Charnes et al. (1978) [32] and Banker et al. (1986) [33] generally date back to 1978 and 1986, respectively, corresponding studies on efficiency measurement of German hospitals can only be found from the year 1985 onwards. Although hospital efficiency has gained an enormous importance in Germany in recent years [6–8], research in this area has stagnated. Table 1 shows the previous research on German hospital efficiency based on the methods DEA or Stochastic Frontier Analysis (SFA). The studies therefore use non-parametric and parametric methods as estimation procedures. In summary, surprisingly, there are hardly any studies on efficiency measurement in German hospitals in the last decade. The studies by Augurzky and Schmitz (2010) [8] and Karmann and Rösel (2016) [34] are the only ones that take into account the differences in hospital care among the German states. Accordingly, no time series data of the German states have been explored since 2013.

**Table 1:** Overview of empirical studies on the efficiency of hospitals in Germany

Author	Basis of analysis	Inputs and outputs / main findings
Taube 1988	613 German hospitals	*Outputs:* patients in different departments *Inputs:* Costs
Helmig 2005	418 German hospitals	*Inputs:* Number of beds, treatment cases per year, sponsorship
Dittrich et al. 2005	105 Saxon and 251 Swiss hospitals	*Inputs:* Number of staff, costs, days of care *Main findings:* Swiss hospitals are less efficient than German hospitals
Staat 2006	160 German hospitals	*Inputs:* daily rates, number of beds *Outputs:* Treatment cases per year, length of stay
Frohloff 2007	1500 German general hospitals	*Inputs:* e.g., ownership *Main findings*: private and non-profit hospitals are on average less efficient than public hospitals
Herr 2008	1500 German hospitals (Data from the years 2000 to 2003)	*Inputs:* Number of beds, treatment cases per year, sponsorship *Main findings:* private and non-profit hospitals are less cost-effective and technically less efficient than publicly owned hospitals
Herr et al. 2009	374 German hospitals (Data from the years 2002 to 2005)	*Inputs:* Number of beds, treatment cases per year, ownership *Main findings*: private (for-profit) and (private) non-profit hospitals are less cost-efficient but more profitable than publicly owned hospitals
Tiemann und Schreyögg 2009	1046 German hospitals	*Main findings*: Public hospitals performed significantly better than their private for-profit and non-profit counterparts. A significant positive association between hospital size and efficiency is shown
Augurzky und Schmitz 2010	1865 German general hospitals (Data from the years 2003 to 2008)	*Inputs:* Staff (physicians, nurses, other staff) and material costs *Outputs:* Number of cases weighted with level of severity *Main findings*: Average efficiency increased slightly between 2003 and 2008. However, hospitals are on average 3% points more inefficient than the top 10% hospitals. But there are notable differences at the state level
Herwartz und Strumann 2011	1500 German general hospitals	*Inputs:* Material costs, personnel, number of beds *Outputs:* Treatment cases per year, number of trainees *Main findings*: Improvement of overall efficiency after DRG introduction
Herr et al. 2011	541 German hospitals	*Main findings:* Higher profit efficiency of private hospitals compared to public hospitals—but differences in cost efficiency
Tiemann und Schreyögg 2011	1878 German acute hospitals	*Main findings*: Conversion from public to private ownership resulted in increased efficiency
Lindlbauer und Schreyögg 2014	1239 acute care German hospitals (Data from the years 2000 to 2010)	*Inputs:* Number of staff (physicians, nurses, other staff) and number of beds *Output:* Weighted cases *Main findings*: Efficiency is negatively associated with case-mix specialization, and positively with medical specialization
Lindlbauer et al. 2016b	225 German Public law hospitals (Data from the years 2002 to 2010)	*Inputs:* Number of staff (physicians, nurses, other clinical staff, administrative staff, other nonclinical staff) and cost of medical supplies *Output:* Weighted cases *Main findings*: The results of the difference-in-difference regressions indicate that corporatization has a positive effect on efficiency
Lindlbauer et al. 2016a	830 acute care German hospitals (with or without quality certification) (Data from the years 2000 to 2010)	*Inputs:* Number of staff (physicians, nurses, other clinical staff, administrative staff, other nonclinical staff) and number of beds *Output:* the number of treated inpatient cases (weighted cases) *Main findings*: The national standard KTQ has significant positive effects on efficiency. The international standard ISO 9001 has a significant negative impact on efficiency
Karmann and Rösel 2016	State-level aggregates of the 16 states (Bundesländer) (Data from the years 1993 to 2013)	*Inputs:* physicians, nurses, and other staff *Outputs:* number of discharges, a quality index, and the quality-adjusted number of discharges *Main findings:* The influence of policy decisions on TFP growth is higher in quality improvement than in increases in input or input amounts. However, hospital policy also depends strongly on the respective reimbursement schemes
Pross et al. 2018	1100 stroke treating German acute care hospitals (Data from the years 2006 to 2013)	*Inputs:* Resource inputs (physicians and nurses) *Outputs:* Risk-adjusted patient volume (stroke-unit) *Main findings*: A conflict of objections is shown between quality improvement and resource reduction. Also, high substantial regional variation in efficiency
Schneider et al. 2020	Emergency cases of 1428 German acute care hospitals (Data from the years 2015 to 2017)	*Inputs:* Number of nurses and physicians, number of beds *Outputs:* In- and outpatient cases *Main findings*: A negative relationship between the urgency score and hospital efficiency is proven. Either a high or low overall urgency score is beneficial. The results indicate that with the medical urgency score hospitals’ efficiency is decreasing

Many scientific research studies use the DEA model in combination with the tobit model to evaluate the technical efficiency of health care and services. The authors of these works solve various problems of microeconomic and macroeconomic nature. These evaluate the technical efficiency of specific organizations (hospitals, clinics, institutes) or only selected medical fields (internal medicine, surgery, psychiatry, etc.) and the influence of selected external parameters (predictors) on the calculated technical efficiency score.

Published studies show that the focus of research on the technical efficiency of general hospitals in the context of exogenous factors is still a current topic. Authors usually test many factors (predictors) as explanatory variables using the tobit model. However, it often turns out that most of the selected factors are not statistically significant in relation to the dependent variable (efficiency score or efficiency trend).

Predictors are expressed using nominal, relative, binary, and scale values. The authors use spatial characteristics [22, 35–37], age, gender, nationality characteristics of the interested population [37], qualification expertise and education of medical personnel [36], size and ownership of hospitals [21–23], health care quality parameters [37, 38] macroeconomic economic and financial characteristics [39–41] specific indices [39].

## Methodology

### Data and methods

The subject of research into the technical efficiency of hospital care is the 16 federal states of Germany (Länder). Each of these states has its own constitution, which reflects the federal, democratic, and social principles of the national constitution, the so-called Basic Law, for its respective state. A key feature of the German political system, which has a particular influence on the health care system, is the division of decision-making authority between the federal government, the states, and civil society organizations. The federal and state governments delegate powers to provide health care services to membership-based and self-regulated organizations, which are referred to as "corporatist bodies" [42, 43]. Thus, Germany has decided for a "self-administration principle" in which neither the state nor the market regulates the complex health care system, but the participants themselves. In the existing economic system of the social market economy, the state sets the framework conditions and tasks for medical care. It issues laws and regulations for this purpose. The market, however, is regulated by the participants [12]. Furthermore, the "system duality" between statutory and private health insurance for primary and mandatory coverage is another key feature of the German health care system. Around 11% of the population is fully insured with a private health insurance company; among OECD countries, only Chile has a similar mixed system between public and compulsory private health insurance [44].

Selected inputs, outputs and exogenous factors are calculated for each year of the six-year period 2015–2020. Each state enters the evaluation 6 times; therefore 96 Decision Making Units (DMUs, 16 × 6) are determined. The logic behind the designation of states as DMUs is as follows: the states are sorted alphabetically by name, so number 1 is Baden-Württemberg, number 2 is Bayern, … the year designation defines a specific DMU, ee.g.,1_2015 (Baden-Württemberg in 2015), 1_2016 (Baden -Württemberg in 2016), see Attachments.

Three inputs (× 1, × 2 and × 3) and one output (y1) were used to estimate technical efficiency and its decomposition. The selected inputs show the most important capacity (Number of beds, × 1) and production factors (Number of physicians × 2, Number of nurses and non-physician staff, × 3) determining both the scope of offer and the fixed costs of healthcare. The selected output is defined by Number of bed occupancy days (y1), which demonstrates the extent of healthcare actually delivered. Realized care is a driver of hospital revenues. The values of inputs and outputs are aggregated for all hospitals in a given state and expressed in relative terms—per 1000 inhabitants, (see Table 2). The choice of these inputs and outputs is based on the needs of this research and their importance is confirmed by the methodologies of previous work (see Table 1).

- ▪ × 1 Number of beds per 1000 inhabitants of a state in a given year (source: [45])
- ▪ × 2 Number of physicians per 1000 inhabitants of a state in a given year (source: [46])
- ▪ × 3 Number of nurses and non-physician staff per 1000 inhabitants of a given state (source: [46])
- ▪ y1 Number of bed occupancy days per 1000 inhabitants of a given state (source: [47])

Two factors were selected on the basis of the verification selection of exogenous factors for regression analysis, in terms of statistical significance and explanatory power.

- ▪ HosN number of all general hospitals (excluding psychiatric institutions and day care) in a given state; (data source: [47])
- ▪ PD Number of inhabitants per km.^2^ (population density of a state) (data source: [48])

HosN characterises the relative level of distribution of health care in a given state. PD indicates a relative limit to the efficiency of health care (population density affects the rate of distribution of health care in a given state). In addition to the above (selected), the number of patients was considered when distinguishing ownership (public, private, non-profit, private), distinguishing states according to the eastern and western sectors (Germany before 1990). However, the significance of these factors was not confirmed in the context of the model studied.

**Table 2:** Basic statistical characteristics of inputs, outputs, and exogenous factors (period 2015–2020)

	** × 1**	** × 2**	** × 3**	**y1**	**HosN**	**PD**
Minimum	4.21	1.74	8.74	1,016.13	12.0	68.9
Maximum	7.39	3.65	17.32	2,136.50	298.0	4,114.8
SD	0.83	0.43	1.91	269.62	85.3	1,063.6
Mean	5.80	2.30	12.25	1,595.22	99.3	683.3
Median	5.77	2.20	11.92	1,647.68	69.5	213.3

Average number of hospital beds (× 1) per 1 thousand inhabitants in the states in the monitored period is 5.8. The lowest number of beds 4.21 per 1 thousand inhabitants was Baden-Württemberg in 2020. The highest number of beds was 7.39 in Bremen (city state) in 2016. The average number of doctors (× 2) in hospitals per 1 thousand inhabitants in the states in the monitored period is 2.30 (lowest 1.74—Niedersachsen in 2015, highest 3.65 Hamburg in 2020). The average number of nurses and medical professionals (× 3) is 12.25 (lowest 8.74—Brandenburg in 2014, highest 17.32—Hamburg in 2020). The average number of occupied bed days (y1) was 1595.22 per 1 thousand inhabitants (lowest number of days 1016.13 in 2020 in Baden-Württemberg, highest number of days 2136.5 Hamburg in 2015).

The largest number of hospitals was in the state of Bayern (298 in 2015, 284 in 2020) and the population density in Bayern was 186 inhabitants per km^2^. The city state of Bremen had the lowest number of hospitals (12 in all years) and the population density in the state of Bremen was 1,623 inhabitants per km^2^. The highest population density was in the city state of Berlin at 4,115 inhabitants per km2 (76 hospitals in 2020) and the lowest density was in the state of Mecklenburg-Vorpommern at 69 inhabitants per km^2^ (33 hospitals in 2020).

A necessary prerequisite for calculating the degree of efficiency according to the DEA model is the correlation between the variables, which, however, should not be higher than 0.8 between the inputs. Otherwise, the inputs are interchangeable.

Correlation coefficients between pairs of variables were calculated according to Pearson's correlation (r) and verified at the 1% level of significance. Only positive correlations were detected, i.e., that as the value of one variable increases, the value of the other variable increases, as shown in the correlation matrix Table 3. A very strong correlation between the input number of beds (× 1) and the output number of days of occupied beds is logical and was expected. Other correlations are optimal and support the assumption of appropriately chosen inputs for multi-criteria evaluation.

**Table 3:** Correlation matrix

	× 1	× 2	× 3	y1
× 1	1	0.688^a^	0.592^a^	0.910^a^
× 2	0.688^a^	1	0.761^a^	0.682^a^
× 3	0.592^a^	0.761^a^	1	0.549^a^
y1	0.910^a^	0.682^a^	0.549^a^	1

^a^the level of significance at 1%

**Fig. 1:**
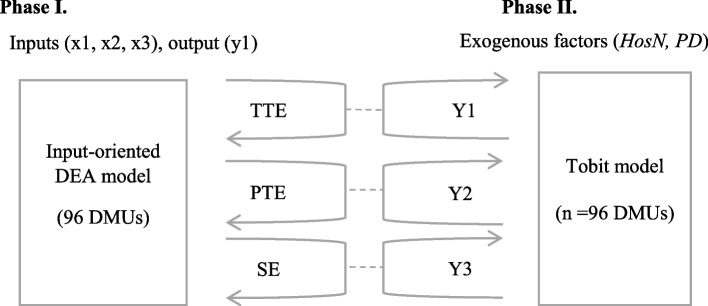
Scheme of two-phase analysis

The research is based on a two-phase analysis including multi-criteria estimation of technical efficiency and regression analysis. The logic is presented in the diagram in Fig. 1. Technical efficiency and its decomposition are estimated according to the input-oriented DEA model.

The approximation of two exogenous factors—the number of hospitals on the territory of a given state (HosN), the number of inhabitants of a state per km^2^ (PD) to the estimated efficiency values—total efficiency Y1, net efficiency Y2 and efficiency from the range Y3 is realized according to the multiple regression analysis of the tobit model.

### Input-oriented DEA model and its decomposition

From the perspective of application, the DEA model is considered to be a universal assessing tool, which means that it can be used, on condition of homogeneity of decision-making units, in the production sector as well as in the sector of services of profit-making and non-profit-making nature. Homogenous decision-making units (DMUs) are created by such set of units that are occupied with the production of identical or equivalent effects, which are denoted as outputs of these units [49].

Estimating efficiency using the DEA model can be implemented both in terms of input orientation and output orientation. An input-oriented model was chosen for the purposes of this investigation.

The calculation of efficiency according to the CCR model is performed with the use of the Charnes-Cooper’s transformation and converted from linear-fractional programming into a standard programming task. The CCR model assumes constant returns to scale (CRS).

The calculation of efficiency according to the BCC model has one additional variable in its objective function (in comparison with the CCR), which corresponds with the restricting condition – condition of convexity, and which will not be restricted by conditions of non-negativity. The BCC model assumes variable returns to scale (VRS).

If the z value equals one, the DMU is efficient. For inefficient units, it applies that their degree of efficiency is lower than one, i.e., e < 1.

The degree of efficiency, which is calculated according to the CCR and BCC models, is a basis for the calculation of the so-called scale efficiency (SE) according to the formula (2). Cooper et al. (2007) [50] define the scale efficiency as the ratio of the degree of efficiency of a decision-making unit gained by the CRS ${\theta}_{CRS}^{*}$ and the VRS ${\theta}_{VRS}^{*}$ model, where the degree of the decision-making unit’s SE is lower or equal to one. The formula (1) stated below, considers the orientation on the inputs, whilst the same indicator and procedure can be applied in case of the orientation on the outputs.

1 $$\mathrm{SE}=\frac{{\theta^\ast}_{CRS}}{{\theta^\ast}_{VRS}}$$

The decomposition of the efficiency (2) allows to express the so-called pure efficiency (PTE) and the scale efficiency (SE).

2 $$\mathrm{CRS} {{\theta }^{*}}_{CRS}= {{\theta }^{*}}_{VRS}\times SE$$

The above-stated facts show that the degree of efficiency calculated by the CRS model is being noted as the total technical efficiency (TTE), and the degree of efficiency calculated by the VRS model as the pure technical efficiency (PTE). This specific decomposition explains the sources of inefficiency, thus whether the cause of inefficiency lies in the operation (pure technical efficiency), or in unfavourable conditions (scale efficiency), or in both [17].

The tobit model takes into account the fact that in the case of the dependent variable one works with limited data [51]. For the application of the model, the assumptions of homoscedasticity and normality of the model for the latent variable are essential. The tobit model is currently one of the most used applied approaches in connection with the DEA model, [see 35] states that "efficiency scores must lie between 0 and 1 or equal 0 or 1. There are usually several values at 1, but often none at or close to 0.”

The tobit equation may be expressed as follows (3);

3 $$\begin{aligned}y_i^*= x_i^{\prime} \beta+ \upmu_{\acute{\imath}}\\ {y}_{i}^{*}>0 if {y}_{i}= {y}_{i}^{*}\\ {y}_{i}^{*}\le 0 if {y}_{i}= 0 \end{aligned}$$

where *x*_*i*_ is the observed independent variable for all situations, *y*_*i*_ is the latent dependent variable limited by values equal to, greater or smaller than 0. *β* shows estimative factors and μ_i_ shows the error (destructive) term. In the equation, the destructive term is expressed as (4):

4 $$\mu \sim \left[0,{\sigma }^{2}\right]$$

That is, the destructive term has zero mean with normal distribution and same variance. Expression of the destructive term in this way also requires expression of the latent variable (y^*^) in the same way *y*^*∗*^ ~ [*0, σ*^*2*^]. In a censored model, “upper censoring” tobit model is recommended or the conditions with the upper limit 1 (as in the efficiency scores) may be expressed as follows with y expressing the values of the observed variable. The tobit model may be defined sometimes as censored from below or above, that on the minus and plus side.

5 $$\begin{aligned}&y_i^\ast=x_i^\ast\beta+\mu_i\\&y=\left\{\begin{array}{c}y_i^\ast,\;if\;y_i^\ast>0\\0,\;if\;y_i^\ast\leq0\end{array}\right.\end{aligned}$$

The model is set as below (6):

6 $$Y=\beta {X}_{i}+{\varepsilon }_{\acute{i} , }Y\ge 0$$

Efficiency scores obtained from DEA in the first stage are used as a dependent variable in the second stage (the tobit model) to allow for the restricted range of efficiency values.

The tobit regression model is assumed as below:

7 $$Efficiency= {\alpha }_{0}+{\alpha }_{1}HosN+{\alpha }_{2}PD$$

## Results: decomposition of the input-oriented DEA model

The calculation of technical efficiency according to the input-oriented DEA model includes three components (TTE, PTE and SE), see Table 4.

**Table 4:** Overall technical efficiency results

	CRS—TTE		VRS – PTE – net t.eff		SE	
	No. Eff	Mean	No. Eff	Mean	No. Eff	Mean
**96 DMUs (2015–2020)**	**5**	**0.8985**	**15**	**0.9471**	**5**	**0.94930**
16 DMUs (2015)	2	0.9348	6	0.9745	2	0.95955
16 DMUs (2016)	3	0.9358	6	0.9719	3	0.96313
16 DMUs (2017)	0	0.9246	0	0.9600	0	0.96359
16 DMUs (2018)	0	0.9133	2	0.9553	0	0.95693
16 DMUs (2019)	0	0.9011	2	0.9426	0	0.95714
16 DMUs (2020)	0	0.7814	1	0.8780	0	0.89544

The average total technical efficiency (TTE) score calculated under CRS assuming constant returns to scale was 0.899. The achieved efficiency rate can also be interpreted as 90% average efficiency. Only five DMUs were fully efficient (e = 1; 100%). Breaking down the results of the calculation by year shows that, on average, states achieved the best results in 2015 (94%) and 2016 (94%) and the worst average results in 2020 (78%).

The average net technical efficiency (PTE) score calculated according to VRS assuming variable returns to scale was 0.947 (i.e., 95%). 15 DMUs were fully effective. Also, as with TTE, the breakdown of PTE results by individual years shows that the best results were achieved at the beginning of the monitored period and the worst results came in 2020.

The average scale efficiency score (SE) calculated according to relation (2) was 95%, fully efficient units were 5. The breakdown of the calculation results by individual years shows that on average the states achieved the best results in the years 2015–2019, correspondingly in the amount of 96%. Deterioration in efficiency from the scale was in 2020, by an average of 6%.

The results according to DMUs are in the appendix (Table 8). Berlin (city state), Brandenburg, Saarland and Hamburg (city state) had the best TE results on average. On average, the worst results were achieved by the states of Bavaria, Baden-Württemberg and Rheinland-Pfalz.

The decomposition results confirm that:

- the average level of technical efficiency of hospital care in the federal states is high (90%) and decreased in the monitored period between 2015 and 2020;
- the deterioration of the average overall technical efficiency was affected by the results of technical efficiency in 2020 and partly in 2019;
- the deterioration of average overall technical efficiency was affected by both net efficiency (use of inputs) and scale efficiency;
- the lower efficiency of two inputs: × 1 (number of beds) and × 2 (number of doctors) contributed most significantly to the deterioration of efficiency.

The results of the analysis of the overall technical efficiency indicate that considering the achieved outputs, inefficient units should reduce the number of beds (by 15% on average) and the number of doctors (by 5% on average). However, the mentioned recommendation is only theoretical, as it can be assumed that the deterioration of the efficiency of hospital care was caused by the COVID-19 pandemic, which necessitated the reduction of normal hospital care and the resolution of the pandemic.

## Results: exogenous factors

Regression analysis according to the tobit model verified whether the measures of technical efficiency that appear in the model as dependent variables Y1(TTE), Y2(PTE) and Y3(SE) of hospital care in individual federal states are influenced by two exogenous factors (independent variables). The first exogenous factor is the HosN number of hospitals in individual states (includes all types of hospitals according to ownership: public, private, non-profit, and private), and the second exogenous factor is the population density of PD countries (number of inhabitants per km^2^).

The results of the tobit regression analysis of the overall technical efficiency of hospital care Y1 are in Table 5.

**Table 5:** Tobit analysis of Y1

Y1	Coef	Std.Err	t	*p* >|t|	[95% Conf. Interval]	
*HosN*	-0.0002	0.0001	-2.37	0.020**	-0.0003	0.0001
*PD*	0.0001	5.9e-06	2.96	0.004**	5.77e-6	0.0001
cons	0.9038	0.0108	83.14	0.000***	0.8823	0.9254
var(e.y)	0.0037	0.0005			0.0027	0.0028

*Prob* > *chi2* = *0.0004; Pseudo R2* =—*0.0637; Log likelihood* = *132.8393; N* = *96*

^***^, **, *the level of signifikance at 1%, 5% and 10%

The results of the tobit analysis show that exogenous factors (HosN, PD) explain the rate of overall efficiency of 6.37% (pseudoR2). If the number of hospitals increases by 1, the overall efficiency decreases by 0.0002 while all other variables are held constant in the model. If the population density increases by 10 inhabitants/km^2^ of inhabitants, then the overall efficiency increases by 0.001 while all other variables are constant in the model.

The results of the tobit analysis show that explaining the level of net technical efficiency (Y2) with the help of exogenous factors cannot be confirmed, the calculations are not supported by statistical significance, see Table 6.

**Table 6:** Tobit analysis of Y2

Y2	Coef	Std.Err	t	*p* >|t|	[95% Conf. Interval]	
*HosN*	- 0.0003	0.0001	0.47	0.638	-0.001	-0.0002
*PD*	6.54e-06	5.19e-06	1.26	0.210	-3.76e-06	0.0002
cons	0.9395	0.0095	98.36	0.000	0.9205	0.9585
var(e.y)	0.0028	0.0004			0.0012	0.0038

*Prob* > *chi2* = *0.4394; Pseudo R2* =—*0.0057, Log likelihood* = *145.2598, N* = *96*

The results of the tobit analysis show that exogenous factors (HosN, PD) can explain the efficiency score from the range of 7.05% (pseudoR2). If the number of hospitals increases by 1, the overall efficiency decreases by 0.0002 while all other variables are held constant in the model. If the population density increases by 10 inhabitants/km^2^, the overall efficiency increases by 0.01 while all other variables are constant in the model, see Table 7.

**Table 7:** Tobit analysis of Y3

Y3	Coef	Std.Err	t	*p* >|t|	[95% Conf. Interval]	
*HosN*	-0.0002	0.0001	-3.63	0.000***	-0.0003	0.0001
*PD*	0.0001	4.73e-06	2.40	0.018**	1.98e-06	0.0000
cons	0.9628	0.0087	110.54	0.000***	0.9455	0.9801
var(e.y)	0 .000774	0.000112			0.0017	0.0031

*Prob* > *chi2* = *0.0000; Pseudo R2* =—*0.0705; Log likelihood* = *154.1168, N* = *96*

The results of the regression analysis according to the Tobit model confirm that:

- the change in the efficiency score can be explained by the influence of exogenous factors only from 6.4% for overall efficiency and from 7.1% for scale efficiency;
- the influence of exogenous factors on the net technical efficiency score cannot be statistically confirmed;
- growth in the number of hospitals worsens the efficiency score (+ 1 hospital will reduce the TTE and SE score by 0.002%);
- the growth of the population density improves the efficiency score (+ 10 inhabitants per km^2^ improves the TTE and SE score by 0.01%).

## Discussion and conclusion

The research looked at the technical efficiency of hospital care at the regional level in the Federal Republic of Germany, examining the aggregated inputs, outputs and exogenous factors of all 16 German states from 2015 to 2020. The analysis of the technical efficiency calculation according to the input-oriented DEA model confirmed that the overall technical efficiency (CRS) of hospital care is relatively high at 90% on average. In response to research question RQ1, it can be answered that the differences in efficiency rates between the different components of overall technical efficiency to explain the causes of technical inefficiency are not clear. Lower efficiency of the inputs × 1 (number of beds) and × 2 (number of physicians) contributed the strongest to the deterioration of efficiency. In essence, the results of the technical efficiency measure detect, however, a source of inefficiency that is highly questionable in the healthcare setting.

Reducing hospital bed capacity makes economic sense if it is accompanied by a reduction in associated costs—personnel, technological and operational—which cannot be applied across the board to all types of hospitals without ensuring their availability and resilience to the surge needs of society. A certain degree of hospital bed vacancy—spare capacity, even at the cost of reduced efficiency—is necessary in terms of continuity of care and optimal accessibility, especially in systems that base public health care on welfare state principles. It is therefore necessary for the supply of hospital care in a given region to include an optimal mix of medical services and capacities. Augurzky et al. [52] state that the German hospital sector needs a reform, regardless of the experience of the period 2019–2021. Slowik, Hentschker [53] state that if the observed trend of decreasing hospital care utilization is maintained, the current hospital structures (number, size and legal forms) can no longer be maintained. Already before the pandemic, changes towards a more demand- and quality-driven structure of the hospital sector were discussed.

Published works [9, 16] show that the orientation towards reduction of human resources in health care for the sake of increasing efficiency can lead to a reduction in the quality of care provided. Which in general could lead to a reduction in the resilience of the health system to surges and unpredictable demands for health care in a given region.

The technical efficiency results also show that the worst average technical efficiency scores were recorded in the states of Germany in 2020, at 78%. Thus, an effect of the COVID 19 pandemic on the technical efficiency scores of hospital care in the states of Germany can be detected, as predicted by research question RQ2. This phenomenon clearly confirms that elective healthcare was subdued at the time of the pandemic. It can also be considered that the decline in the efficiency of hospital care in Germany could actually have been higher in 2020 if state intervention had not been implemented. Augurzky et al. [52] report that due to the uncertainty of expected COVID-19 cases, hospitals were expected to keep beds and capacity vacant and expand intensive care capacity from mid-March 2020. Keeping capacity vacant would result in significant revenue losses for hospital operators with only a slight decrease in costs. The German government therefore opted for a comprehensive support package in which revenue losses due to a decrease in the volume of services billed through the DRG system were compensated by compensation payments to offset the financial imbalance of hospitals.

Regardless of the pandemic, the loss or reduction in hospital efficiency can generally be attributed to the inelasticity of supply in the face of fluctuating demand. Research by Schneider et al. [9] compared the technical efficiency results of selected German hospitals with respect to their urgency score and urgency dispersion conditions. The results of their research confirm that hospitals with a higher urgency score are more efficient and, on the contrary, hospitals where the urgency dispersion is higher, the efficiency decreases. This research highlights that the reduction in efficiency of hospitals is affected by unpredictable, surge and range significant demand. Hospitals are forced to adjust the organization of health care work due to fluctuations in urgent, and less urgent cases. Changing routine procedures then leads to efficiency losses at the organizational level. This line of reasoning could also be applicable to our findings in relation to the COVID-19 pandemic.

Research questions RQ3 and RQ4 focused on the impact of the technical efficiency score on external factors, i.e., the number of general hospitals and population density in the German federal states. The analysis of the tobit regression calculation shows that both external factors under study had an impact on the efficiency scores of health care provided by hospitals in the German states. The states with lower population density and more hospitals showed lower hospital care efficiency scores. However, this effect is small. These findings also indicate the fact that the German federal states, although comprising different sized states including urban states, have a comparatively robust and therefore relatively resilient hospital care system in terms of efficiency and its distribution across the territory.

However, as shown in [31], in regions where the distribution of hospitals in the territory is unbalanced (unilaterally concentrated only in the largest city), there was a failure to manage care for patients affected by COVID-19 during the pandemic in 2019 and 2020. In regions where the need for care for COVID patients was higher than the capacity of the health system, the conflict between health equity and efficiency was shown. Also, the work of Culyer [20] states that in the health system, the following is often true: An inefficient allocation can be equitable. An efficient allocation can be inequitable.

## Figures and Tables

**Fig. 2:**
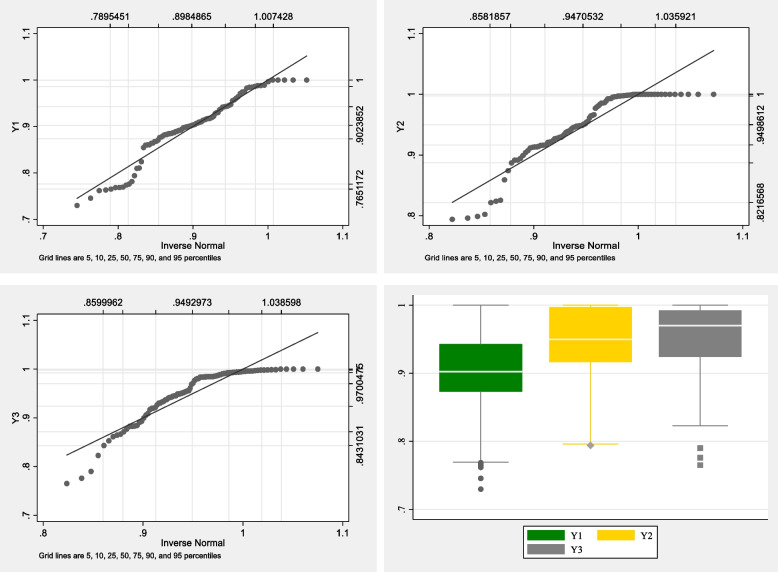
Inverse Normal Analysis and Boxplot Y1, Y2 and Y3, *n* = 96

## Data Availability

Not applicable. The data are given in the sources—references.

## Appendix

Table 8

**Table 8:** Results of the technical efficiency decomposition

**DMUs**		TTE(CRS)	PTE(VRS)	SE	**DMUs**	TTE(CRS)	PTE(VRS)	SE
Baden-Württemberg	1_2015	0.870	0.986	0.883	1_2018	0.864	1.000	0.864
Bayern	2_2015	0.882	0.941	0.937	2_2018	0.861	0.934	0.921
Berlin (city state)	3_2015	0.996	0.998	0.999	3_2018	0.986	0.997	0.989
Brandenburg	4_2015	1.000	1.000	1.000	4_2018	0.971	1.000	0.971
Bremen (city state)	5_2015	0.943	0.981	0.961	5_2018	0.917	0.929	0.987
Hamburg (city state)	6_2015	0.985	1.000	0.985	6_2018	0.944	0.948	0.996
Hessen	7_2015	0.938	0.986	0.951	7_2018	0.902	0.954	0.945
Mecklenburg-Vorpommern	8_2015	0.914	0.916	0.998	8_2018	0.905	0.913	0.991
Niedersachsen	9_2015	0.917	1.000	0.917	9_2018	0.889	0.996	0.893
Nordrhein-Westfalen	10_2015	0.907	0.921	0.985	10_2018	0.898	0.913	0.983
Rheinland-Pfalz	11_2015	0.899	0.966	0.930	11_2018	0.893	0.948	0.942
Saarland	12_2015	1.000	1.000	1.000	12_2018	0.975	0.996	0.979
Sachsen	13_2015	0.961	0.965	0.995	13_2018	0.942	0.948	0.993
Sachsen-Anhalt	14_2015	0.917	0.932	0.984	14_2018	0.889	0.894	0.994
Schleswig–Holstein	15_2015	0.882	1.000	0.882	15_2018	0.877	1.000	0.877
Thüringen	16_2015	0.946	1.000	0.946	16_2018	0.900	0.914	0.985
Baden-Württemberg	1_2016	0.876	0.993	0.882	1_2019	0.861	0.999	0.861
Bayern	2_2016	0.883	0.945	0.934	2_2019	0.864	0.940	0.919
Berlin (city state)	3_2016	1.000	1.000	1.000	3_2019	0.983	0.999	0.984
Brandenburg	4_2016	1.000	1.000	1.000	4_2019	0.964	0.979	0.985
Bremen (city state)	5_2016	0.956	1.000	0.956	5_2019	0.886	0.892	0.994
Hamburg (city state)	6_2016	0.988	1.000	0.988	6_2019	0.936	0.939	0.997
Hessen	7_2016	0.915	0.965	0.949	7_2019	0.895	0.951	0.941
Mecklenburg-Vorpommern	8_2016	0.928	0.929	0.998	8_2019	0.887	0.899	0.986
Niedersachsen	9_2016	0.920	1.000	0.920	9_2019	0.891	1.000	0.891
Nordrhein-Westfalen	10_2016	0.909	0.924	0.984	10_2019	0.892	0.913	0.976
Rheinland-Pfalz	11_2016	0.899	0.946	0.950	11_2019	0.855	0.916	0.933
Saarland	12_2016	1.000	1.000	1.000	12_2019	0.922	0.928	0.994
Sachsen	13_2016	0.957	0.961	0.996	13_2019	0.913	0.928	0.984
Sachsen-Anhalt	14_2016	0.910	0.916	0.993	14_2019	0.885	0.892	0.992
Schleswig–Holstein	15_2016	0.885	0.977	0.905	15_2019	0.884	1.000	0.884
Thüringen	16_2016	0.947	0.993	0.954	16_2019	0.901	0.907	0.993
Baden-Württemberg	1_2017	0.868	0.995	0.871	1_2020	0.765	1.000	0.765
Bayern	2_2017	0.868	0.937	0.926	2_2020	0.746	0.874	0.853
Berlin (city state)	3_2017	0.989	0.998	0.991	3_2020	0.860	0.917	0.938
Brandenburg	4_2017	0.988	0.992	0.996	4_2020	0.824	0.951	0.867
Bremen (city state)	5_2017	0.930	0.944	0.986	5_2020	0.794	0.796	0.997
Hamburg (city state)	6_2017	0.974	0.984	0.990	6_2020	0.811	0.824	0.984
Hessen	7_2017	0.911	0.956	0.953	7_2020	0.769	0.912	0.843
Mecklenburg-Vorpommern	8_2017	0.918	0.922	0.996	8_2020	0.776	0.822	0.944
Niedersachsen	9_2017	0.907	0.998	0.909	9_2020	0.774	0.997	0.776
Nordrhein-Westfalen	10_2017	0.904	0.922	0.981	10_2020	0.768	0.826	0.931
Rheinland-Pfalz	11_2017	0.879	0.927	0.949	11_2020	0.730	0.887	0.823
Saarland	12_2017	0.984	0.986	0.998	12_2020	0.763	0.799	0.955
Sachsen	13_2017	0.942	0.949	0.993	13_2020	0.810	0.859	0.942
Sachsen-Anhalt	14_2017	0.903	0.904	0.999	14_2020	0.762	0.802	0.950
Schleswig–Holstein	15_2017	0.897	0.997	0.900	15_2020	0.781	0.989	0.790
Thüringen	16_2017	0.930	0.948	0.980	16_2020	0.769	0.794	0.969

## Abbreviations

CRS: Constant returns to scale
DEA: Data Envelopment Analysis
DMUs: Decision Making Units
DRG: Diagnosis-related group
HosN: Number of hospitals
RQ: Research questions
PD: Population density of the state
PTE: Pure technical efficiency
SE: Scale efficiency
TTE: Total technical efficiency
VRS: Variable returns to scale
